# Nanomaterial-Integrated Cellulose Platforms for Optical Sensing of Trace Metals and Anionic Species in the Environment

**DOI:** 10.3390/s21020604

**Published:** 2021-01-16

**Authors:** Carlos Bendicho, Isela Lavilla, Francisco Pena-Pereira, Inmaculada de la Calle, Vanesa Romero

**Affiliations:** Centro de Investigación Mariña, Universidade de Vigo, Departamento de Química Analítica y Alimentaria, Grupo QA2, 36310 Vigo, Spain; isela@uvigo.es (I.L.); fjpena@uvigo.es (F.P.-P.); incalle@uvigo.es (I.d.l.C.); vromero@uvigo.es (V.R.)

**Keywords:** cellulose substrates, sensors, PADs, microPADs, metal ions, anionic species, environmental samples

## Abstract

The development of disposable sensors that can be easily adapted to every analytical problem is currently a hot topic that is revolutionizing many areas of science and technology. The need for decentralized analytical measurements at real time is increasing for solving problems in areas such as environment pollution, medical diagnostic, food quality assurance, etc., requiring fast action. Despite some current limitations of these devices, such as insufficient detection capability at (ultra)trace level and risk of interferent effects due to matrix, they allow low-cost analysis, portability, low sample consumption, and fast response. In the last years, development of paper-based analytical devices has undergone a dramatic increase for on-site detection of toxic metal ions and other pollutants. Along with the great availability of cellulose substrates, the immobilization of receptors providing enhanced recognition ability, such as a variety of nanomaterials, has driven the design of novel sensing approaches. This review is aimed at describing and discussing the different possibilities arisen with the use of different nanoreceptors (e.g., plasmonic nanoparticles, quantum dots, carbon-based fluorescent nanoparticles, etc.) immobilized onto cellulose-based substrates for trace element detection, their advantages and shortcomings.

## 1. Introduction

Conventional analytical methodology using equipment in central labs is well established but a number of stages such as sampling, sample preparation, and optimization of instrumental parameters need to be accomplished before analytical information can be obtained. For instance, in the control of environmental pollutants, time-consuming operations such as sampling at fixed times and locations, sample preservation procedures and storing need to be typically carried out prior to analysis. Generally, centralized analytical equipment involves high acquisition and maintenance costs and besides, it requires special facilities for suitable operation and well-trained technical personnel. In addition, most atomic spectrometry techniques that are suitable for trace element analysis involve high energy and sample consumption as well as the need for gases.

Miniaturization of analytical systems plays an important role in the development of novel strategies for on-site analysis, besides contributing to decrease the reagents and samples consumption, and ultimately, energy, in agreement with the current trends of green chemistry [1]. Total analysis systems (TAS) or lab-on-chip has been a first advance in this direction [2]. The immobilization of a variety of receptors (recognition elements) on solid substrates are suitable strategies for designing novel analytical systems, as reflected in the literature. Thus, immobilization of nanomaterials based on metals and oxides on different surfaces, i.e., glass, quartz, vitreous carbon, metals, porous polymer monoliths, etc., is a necessary requirement for surface plasmon resonance sensors, surface enhanced Raman dispersion, electrochemistry, microextraction, analytical separations and quartz crystal microbalance sensors [3].

Implementation of biodegradable substrates such as cellulose-based materials of widespread use, has enabled the development of a large variety of microfluidic analytical systems with applications mainly for medical diagnosis [4]. Unlike lab-on-a-chip devices relying on silicon, glass, or polymers such as polydimethylxilosane (PDMS), which require complicated fabrication processes, paper-based microfluidic devices, also known as lab-on-a-paper, are based on cellulose-based materials. Cellulose is a biopolymer that can be found in nature, e.g., cells in wood and cotton [5,6]. This biopolymer is formed by linear homo-polysacaride based on D-anhidroglucopyranose units linked by beta-1-4-glycosidic bonds. In agreement with the World Health Organization (WHO), clinical diagnosis carried out with paper-based analytical devices fulfils the terms included within the acronym ASSURED (affordable, sensitive, specific, user-friendly, rapid and robust, equipment free and deliverable to end-users) [4].

At this point, it is necessary to distinguish between two main configurations in which cellulose-based analytical devices are presented, namely, paper-based microfluidic devices (microPADs) and paper-based assay devices (PADs). The first ones are formed by a fluidic network on the paper substrate where sample and reagent transport occur by capillarity. These systems have evolved from the lateral flow simple approaches up to more complex tridimensional designs. Commonly, the acronym PAD (paper-based analytical device) is employed to cover both possibilities, but in this review, both acronyms will be kept so that we can maintain the difference between them. Paper-based assays (also known in the literature as paper-based sensors or spot tests) have their origin in the old classical qualitative analysis firstly proposed by F. Feigl, where many cations and anions could be detected by different colorimetric or fluorimetric reactions. Many reactions were carried out on filter paper, taking advantage of the great porosity and adsorption ability which enables to accomplish assays with enhanced sensitivity in comparison with other substrates [7].

The interest in the development of paper-based analytical devices relies on the hydrophilic nature of the substrate, easy construction and handling, low cost, high capillarity (wicking effect), and porosity which facilitates the immobilization of receptors and analyte diffusion, disposable use, biodegradability, and biocompatibility. In addition, white color is excellent for colorimetric transduction and no external forces are required to conduct the analyte up to the detection area.

**Figure 1:**
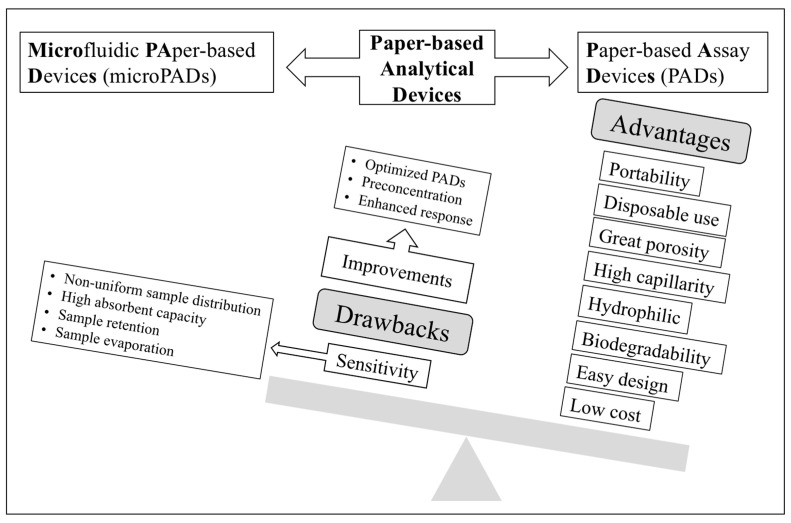
Schematic diagram showing the advantages and drawbacks of paper-based analytical devices as well as possible pathways for enhancing sensitivity.

Human exposure to metal ions contributes to morbidity and mortality, particularly in countries that lack strict regulations. Likewise, some anionic species can also cause toxic effects, so their presence in waters must be controlled. To ensure water quality after purification processes in wastewater treatment plants, suitable analytical control of several parameters including several metal ions (e.g., Cd, Cu, Cr, Hg, Pb, Ni, Zn, and As) as well as anions (chloride, sulphate, nitrate, nitrite, sulphide, iodide, fluoride, cyanide, etc.) is required [8]. Drinking water regulations also include maximum concentration levels for several metal ions and anions, which cause harmful effects on environment and health [9,10,11]. The different guidelines show the need for very sensitive techniques for successful detection in drinking water in the range of ppm-ppb. Despite the above-mentioned advantages accounting for the sharp increase in the development of PADs and microPADs for determining trace element analysis in environmental samples, lack of enough sensitivity to fulfil most drinking water guidelines is a main drawback, something that could be alleviated by further progress in three areas (Figure 1), i.e., introduction of receptors providing enhanced response (e.g., nanostructured receptors), optimized designs, and integration with preconcentration approaches, as will be discussed in this review.

The combination of paper-based analytical devices with information and communication technologies (ICTs) such as smartphones, scanners, digital cameras, etc., can drive the design of green, miniaturized, low cost, and easy-to-use technologies for detection of elemental species.

**Figure 2:**
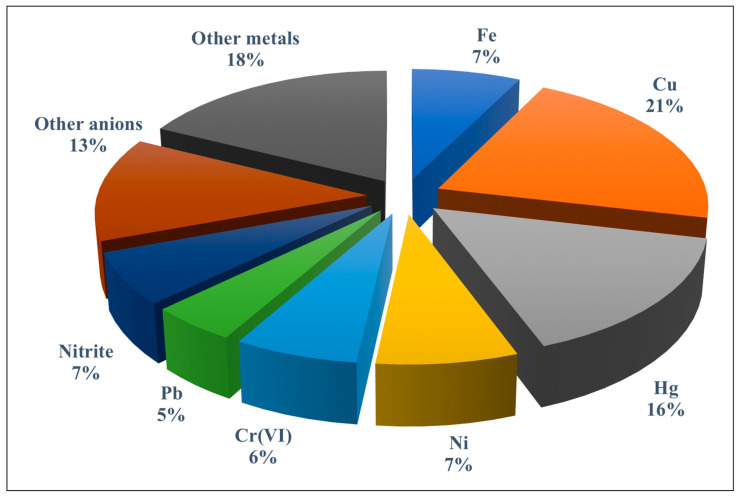
Percentage of publications dealing with paper-based assay devices (PADs) and microfluidic paper-based devices (microPADs) for the detection of trace metals and anions. The group of other metals includes Mn, Zn, Ag, Se, Cd, Co, Ce, Al, and As; the group of other anions includes iodate, iodide, cyanide, fluoride, sulphate, sulphite, chloride, and sulphide.

The role played by paper-based analytical devices as sensing platforms for trace elements using different transduction systems has been discussed in both general reviews [12,13,14,15,16,17,18,19,20,21] and specific reviews [22,23,24] published in recent years. However, no review has addressed the new design of paper-based assay devices where instead of conventional receptors (i.e., chemoreceptors), a variety of nanostructured materials with unique optical properties such as plasmonic nanoparticles (NPs) (e.g., AgNPs and AuNPs), fluorescent nanoparticles (e.g., quantum dots, carbon quantum dots, graphene quantum dots, etc.), and other hybrid materials are implemented. In addition, new sensing approaches that integrate a PAD with a separation/preconcentration technique have arisen during the last few years. Figure 2 shows the percentage of applications reported for metal ions and anions using PADs and microPADs mainly in the environmental field.

In the first part of this review, the state-the-of-art of PADs and microPADs for optical detection (colorimetry and luminescence) of metal ions and anionic species using both traditional chromogenic reagents and nanostructured receptors for analyte recognition is addressed. In the second part, several PADs integrated with preconcentration techniques for improving selectivity and sensitivity are discussed.

## 2. Detection of Metal Ions and Anionic Species by Paper-Based Analytical Devices along with Chromogenic/Fluorogenic Reagents

Most applications reported for the detection of trace metals and anions using chromogenic/fluorogenic receptors has mainly been performed with different types of microPADs (i.e., 2D, 3D, and distance-based).

In microPADs designed for the detection of metal species, analytes are driven by capillary action to a detection area where the recognition event takes place. Colorimetric detection has been typically carried out using a chromogenic reagent such as a complexing agent, giving rise to the formation of metal-ligand colored complexes. The microPADs require hydrophobic barriers to be printed on the substrate to direct the flow. The suction of a liquid by a capillary pressure caused by the curvature of the meniscus is a fundamental mechanism that explains the two-phase flow in porous media. The flow in these systems is laminar because of the size <20 µm of fibers and pores associated, resulting in low Reynolds’ numbers. In the modeling of these flows, the law of Darcy and the equation of Lucas–Washburn are fulfilled under certain premises [21]. Different techniques such as ink printing, wax printing, serigraphic printing, or even the use of a marker of permanent ink, among others, have been used for printing the hydrophobic barriers [24].

Representative applications of PADs and microPADs both using chromogenic/fluorogenic reagents [25,26,27,28,29,30,31,32,33,34,35,36,37,38,39,40,41,42,43,44] and nanostructured materials [45,46,47,48,49,50,51,52,53,54,55] for the detection of metal ions in environmental samples are shown in Table 1. 

In the last years, different chromogenic reagents such as Cs(I)-chrysoidine [25], Cu(II)-porphyrin derivative [26], Cu(II)-diethyldithiocarbamate [27], Cd(II)-cadion [27], Ni(II)-dimethylglyoxime [27], Cr-diphenylcarbazide [27,28], Cu(II)-bathocuproine [29], etc., have been proposed. The use of nonselective receptors such as 4-(2-pyridylazo) resorcinol (PAR) for the detection of elements like Mn(II) and Co(II) requires masking strategies for improving the selectivity [30]. The catalytic effect caused by Cu(II) on the decomposition of S-nitrosothiols (RSNO) brings about a color change, which can be used for colorimetric sensing of this metal ion onto a PAD by means of a smartphone [31].

**Figure 3:**
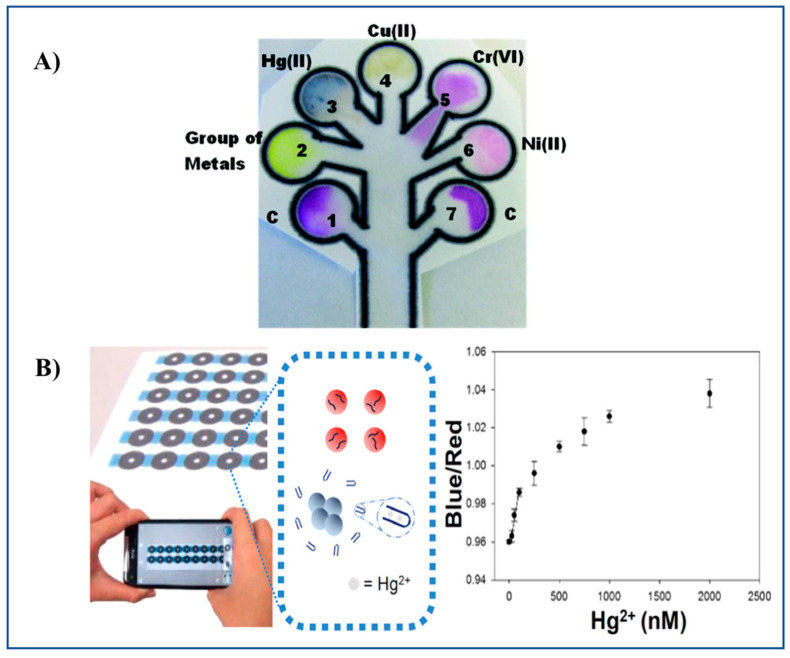
Representative examples of a microPAD and a PAD. (**A**) 2D microPAD for multiplexed detection of Hg(II), Cu(II), Cr(VI), and Ni(II) using Zincon, sodium diethyldithiocarbamate, 1,5-diphenylcarbazide and dimethylglyoxime as chromogenic reagents, respectively. The microPAD was employed to detect individual metal ions or a mixture of metal ions on the basis of an enzyme-based assay (β-galactosidase) [32]. (**B**) Detection of Hg(II) ions by a PAD based on the aggregation of ssDNA-attached AuNPs [47]. Figure 3A,B reproduced with permission of the American Chemical Society [32,47].

Chromogenic reagents such as Zincon, dimethylglyoxime, diethyldithiocarbamate, and diphenylcarbazide have been employed in a microPAD for the multiplexed determination of Hg(II), Ni(II), Cu(II), and Cr(VI) in waters [32] (Figure 3A). Multiplexed determination of Ni(II), Cu(II), and Fe(III) using a microPAD with distance-based detection has also been reported [33].

The limit of detection (LOD) for metal ions in waters achieved with these sensing approaches depends on the paper thickness, detection mechanism, kind of receptor, and size of channels in the microPAD [24].

**Figure 4:**
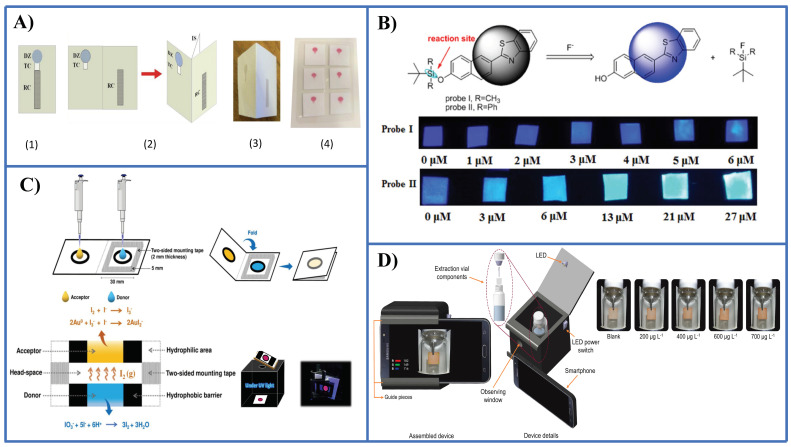
Representative examples of PADs and microPADS for the detection of anions. (**A**) Schematic diagrams showing 2D (sub-figure 1) and 3D (sub-figures 2,3) microPADs for the detection of nitrite and nitrate. Sub-figure 4 shows a card with six 3D microPADs following detection of nitrite with the Griess reagent. Nitrate was measured after conversion into nitrite with Zn microparticles immobilized in an hydrophilic channel of the microPAD [56]; (**B**) detection of fluoride by a PAD using two ratiometric fluorescent probes based on the Si-O bond cleavage [61]; (**C**) iodate detection by a PAD integrated with a membraneless gas-separation device and BSA-AuNCs as sensing probes. Prior reduction of iodate to free iodine was performed [64]; (**D**) determination of sulphite anion by a PAD integrated in a microextraction system [65]. Figure 4A,C,D is reproduced with permission of the American Chemical Society [56] and Elsevier [64,65].

A few PADs have been reported so far for the detection of anionic species. Nitrite has received much attention, for instance using the Griess reaction [56,57,58], tetrazine-based chemistry [59], and a modified Griess reaction [60]. Nitrate can be easily determined prior to reduction to nitrite followed by the Griess reaction in the PAD [56] (Figure 4A). Fluoride has been determined by means of two probes (namely, probe I and probe II) based on the cleavage of the Si-O bond induced by this anion [61] (Figure 4B). With probe I it is possible to recognize fluorides in the safety level in drinking water (1 ppm), while probe II enables to establish dangerous high levels in water (4 ppm). Sulphate in the form of hydrogen sulphate ($HSO_{4}^{-})$ was determined taking advantage of the amphiphilic properties of this species using covalently anchored rhodamine onto a cellulose surface [62].

A microPAD was described recently by Duangdeewong et al. [63] for the determination of iodate in table salt and irrigation water. Oxidation of hydroxylamine by iodate to yield nitrite which in turn reacts with the Griess reagent is used for sensing. The color is captured with a digital camera giving rise to a LOD of 38.1 ppm with a relative standard deviation (RSD) less than 2%.

Multiplexed detection of metal ions together with other substances has also been reported. Thus, a multiplexed detection of Fe(III), Ni(II), and bovine serum albumin (BSA) has been described by Xiong et al. [42] A microPAD impregnated with different chromogenic reagents was employed. LODs of 0.1 mM (Fe), 0.5 mM (Ni), and 1 µM (BSA) and a repeatability below 3%, were reached.

## 3. Detection of Trace Metals and Anions Using Paper-Based Analytical Devices and Nanostructured Receptors

### 3.1. Plasmonic Nanoparticles Immobilized onto Cellulose Substrates

The surface plasmon resonance (SPR) phenomenon consists of the collective oscillation of electrons located at the conduction band when some metal nanoparticles (e.g., Au, Ag, Cu) are irradiated with light of suitable frequency. It is called local surface plasmon resonance (LSPR), when the dimensions of the metal nanoparticles are less than the wavelength of the radiation [66]. When light is of the same frequency as oscillations, the electron cloud vibrates or resonates, giving rise to absorption. The LSPR depends on size, shape, and composition of the nanoparticles (nanorods and nanoshells are more sensitive to changes in the local refractive index than nanospheres). The absorption band in the spectrum corresponding to the LSPR, is influenced by several factors, being the basis for plasmonic sensors. Thus, the LSPR band can be shifted when the analyte binds the surface of the nanoparticle, which causes a color change, thus allowing colorimetric detection.

Colorimetric assays using plasmonic nanoparticles instead of typical chromogenic reagents may provide enhanced sensitivity and improved detection limits. Plasmonic nanoparticles display better stability as compared to most organic dyes and more importantly, they have higher extinction coefficients, hence leading to better sensitivity.

As shown in Table 1, several kinds of nanostructured receptors including plasmonic NPs have been immobilized onto cellulose substrates to build up a paper-based analytical device. A clear trend observed in the last years when using PADs as compared to microPADs, is the implementation of nanomaterials for optical detection instead of conventional chromogenic/fluorogenic reagents.

Guo et al. [45] used silanization–titanium dioxide modified filter paper (STCP) to trap bovine serum albumin (BSA) capped AuNPs. The BSA-AuNPs anchored onto the STCP were etched by Cr(VI) in the presence of HBr, giving rise to a visible color change. Cr(VI) does not have enough ability to oxidize Au but in the presence of bromide the potential of Au(I)/Au(0) decreased due to the formation of the AuBr_2_^−^ complex. The etching process caused the decrease in particle size and amount of AuNPs onto the filter. The method shows high resistance to interferences with a LOD of 0.28 µM Cr(VI).

A paper-based sensor using AgNPs as receptors was used for the detection of Cu(II) [46]. The sensing mechanism consisted of the decrease in the surface plasmon resonance absorption peak and the formation a new red-shifted peak as a result of AgNPs aggregation that occurs when Cu(II) is present. Both PADs and µPADs were studied, using measurements by naked eye and digital camera. Homocysteine and dithiothreitol bind AgNPs through Ag-S bonds, and then they bind to Cu(II), which in turn results in AgNPs aggregation. A change in the yellow color of AgNPs to green-brown was observed in the presence of Cu(II). The sensor was applied to the determination of Cu(II) in pond and tap water.

Chen et al. [47] developed a PAD for Hg(II) detection in water sources (spiked pond and river water), based on AuNPs thymine-Hg(II)-thymine coordination chemistry (unmodified ssDNA are used as receptors for Hg instead of using thiolated or fluorescent-labeled ssDNA). A LOD of 50 nM Hg can be achieved. A sensing mechanism based on AuNPs aggregation is proposed, which results in a color change from red to purple and then to blue according to the degree of AuNP aggregation. Colorimetric sensing was triggered by adding NaCl to the sample solution along with modified AuNPs, and deposited onto paper for color enhancement. Readout in the PADs was carried out by means of a smartphone. Images were transmitted for cloud computing (Figure 3B).

The need for increased concentrations of the plasmonic NPs in PADs in comparison with assays carried out in solution has been observed [46]. A drawback reported when immobilizing plasmonic NPs onto cellulose is the limited aggregation that may occur, which, in turn, is responsible for changes in color occurring in the presence of the analyte. In some works, the possibility of making the assay in solution with further transfer of the probe to the PAD has been tried [47].

Chen et al. [48] reported a PAD based on the oxidation of the reagent 3,3,5,5-tetramethylbenzidine (TMB)-H_2_O_2_ by PtNPs that are added to the sample. PtNPs cause a fast oxidation of TMB, giving rise to a blue color. Hg(II) interact with PtNPs, inhibiting the oxidation process. The decrease of blue color occurring as a result of the presence of Hg(II) can be observed by naked eye. For low Hg(II) concentration (0.01 µM), a fiber optic sensor device can be employed after capturing images by digital camera or smartphone.

Curcumin nanoparticles (CURNs) in PADs were used as receptors for selective sensing Hg(II) [49]. A LOD of 0.17 ppm (without preconcentration) and 0.003 ppm after 50 times preconcentration was obtained. The yellow color of CURNs onto paper changed to light yellow in the presence of Hg(II). The image of the test zone was captured by a digital camera and processed by Adobe photoshop CS8. A sensing mechanism based on the complex formation between Hg(II) and CURNs was proposed. The assay was applied to the determination of Hg(II) in environmental and industrial water samples. For enhancing sensitivity, repeated additions of sample onto the same test zone were performed.

A paper-based sensor for Hg(II) and NH_3_ was developed following AgNPs phytosynthesis [50]. An aqueous leaf extract of *Convolvulus cneorum* was used for reduction and stabilization of Ag(I) into AgNPs. A LOD of 5 ppb Hg(II) and 5 ppm ammonia for assays carried out in solution was obtained. The sensor enabled the detection of Cr(VI) and aqueous ammonia by naked eye using the PAD. The LSPR band of AgNPs shifted to blue in the presence of Hg(II) and ammonia.

A sensor for the determination of Ni(II) in both solution and PAD format was described by Jun-jie et al. [51]. The reagent Zircon incorporated with ZnSiO_3_ nanospheres was employed as hybrid ionophore. A color change from red to blue further discolors in the presence of increasing Ni(II) concentrations. The selectivity of the assay can be improved adding Na_2_-ethylenediaminetetraacetic acid (Na_2_-EDTA) as co-ionophore. The LOD of the sensor was 36 nM Ni in solution and 83 nM in the PAD. Scanning of PADs and image processing by ImageJ software were used.

Shrivas et al. [52] reported a smartphone-paper based sensor for detection of Fe(III) in water and blood. A solution of Ag modified with cetyltrimethylammonium bromide was used as recognition element. The change in color of AgNPs/cetyltrimethylammonium bromide (AgNPs/CTAB) which occurs in the presence of Fe(III) is measured using a smartphone and ImageJ software. The discoloration observed is explained on the basis of an electron transfer reaction. A LOD of 20 µg/L with a RSD of 3.2% was obtained.

Dong et al. [53] designed a colorimetric paper sensor which relies on belt-like ZnSe nanoframes consisting of numerous ZnSe nancrystals. The sensing mechanism is based on the cation-exchange characteristic of chalcogenides, allowing the detection of Ag(I), Cu(II), and Hg(II). Cu(II) could be detected at a level below 1 ppm whereas for Hg(II) and Ag(I) the detection limit was below 5 ppm.

So far, most paper-based assays were developed following the capture of light reflection (color intensity) using a scanner or camera and further processing of the image with image analysis software. In the transmission mode, a portable transmission densitometer is employed. With this measurement mode, an improvement in the accuracy and sensitivity for detection of high concentrations is achieved. This approach was applied to the determination of Cu(II), Fe(III), and Ni(II), but conventional chromogenic agents were employed [67].

A few papers have addressed the direct determination of anionic species using PADs along with nanomaterials as receptors.

The determination of chloride was carried out on the basis of the oxidative etching of AgNPs in the presence of Cl^−^ and H_2_O_2_ which causes a white precipitate of AgCl [68]. A distance-based microPAD is employed where the length of the white band is proportional to the chloride concentration in the water samples. The LOD reached by naked eye is 2 ppm with a RSD less than 4.51%.

### 3.2. Fluorescent Nanoparticles Immobilized onto Cellulose Substrates

Several fluorescent nanostructured materials have been implemented mostly in PADs for the detection of metal ions. Thus, inorganic quantum dots [69], carbon dots and graphene carbon dots [70], and metal nanoclusters [71] have shown a great potential for designing new sensors so their implementation as receptors for building new assays on cellulose platforms is very promising. Luminescent nanoparticles possess quantum yields much higher than those of conventional organic fluorophores as well as enhanced chemical and photoluminescent stability.

Applications have been mainly focused on Hg(II), Cu(II), and Se(IV). Table 2 shows main applications [72,73,74,75,76,77,78,79,80,81,82,83,84,85,86,87,88,89,90,91,92] where luminescent nanoreceptors are immobilized onto cellulose substrates for sensing trace elements. Direct deposition of the sample onto the PAD is considered here. Assays where the PAD is combined with an enrichment process will be addressed in Section 4.

It is necessary to highlight that in a significant number of papers published, LODs are referred to the application of the probe in solution using fluorimetry.

#### 3.2.1. Quantum Dots

Quantum dots (QDs) are fluorescent nanocrystals made from semiconductor materials, usually of spherical shape and a size less than 10 nm in diameter. QDs display excellent photoluminescent properties [69]. Typical QDs composition includes elements of groups II-VI (e.g., CdSe), III-V (e.g., InAs), and IV-VI (e.g., PbS), and core-shell systems (e.g., CdSe/ZnS).

A paper-based sensor has been reported for visual detection of Hg(II). For this, 2-hydroxyethyldithiocarbamate (HDTC)-QDs were immobilized onto cellulose acetate [77]. A color change due to the high affinity of HDTC on the surface of CdSe/ZnS QDs toward Hg(II) occurs. An orange fluorescence was displayed under 365 nm UV lamp which changes to red in the presence of Hg. The LOD of Hg(II) was 0.2 ppm with this approach.

Inkjet-printed CdTe QDs onto a paper strip were used for speciation of Ag(I) and AgNPs by distance-based readout method [78]. The capillary movement of Ag^+^ up to the strip causes the quenching of the fluorescence from CdTe QDs due to a cation exchange reaction between Ag^+^ and CdTe QDs. The height of the quenched fluorescent band is proportional to the Ag concentration in the solution. However, no quenching occurred in the presence of AgNPs, thus enabling speciation of both species. For the detection of total Ag, a digestion with conc. HNO_3_ was carried out so as to convert AgNPs into Ag^+^. A LOD of 0.01 ppm Ag was obtained. Thus, a disposable and instrument-free method was accomplished for on-site identification and speciation of Ag^+^ and AgNPs.

The formation of CoS_2_ when a paper-based sensors uses ZnS as a receptor is used for detection of Co(II) [88]. The strong absorption in the visibility of CoS_2_ formed a brown color which allows its measurement by a mobile camera and further color processing using image software.

Liu et al. [89] reported a PAD with fluorescent ZnO NPs as probe for the detection of Cu(II). Quenching of the fluorescence occurs in the presence of the analyte. Fluorescence images were taken by a mobile phone camera following irradiation with an UV lamp. A detection range of 10–1000 µM is obtained.

#### 3.2.2. Carbon Quantum Dots and Graphene Quantum Dots

Carbon quantum dots (CQDs), also known as carbon dots (CDs), are fluorescent spherical NPs made of carbon with a diameter less than 10 µm. Unlike inorganic QDs, carbon-based fluorescent nanomaterials can be easily synthesized from nontoxic precursors and under green synthetic methods. In addition, they are highly soluble in aqueous media. Although their quantum yield is lower than that of inorganic QDs, it can be improved by suitable doping. Graphene quantum dots (GQDs) are fluorescent π-conjugated single sheets (i.e., disk of graphene in the size range of 2–20 nm). Both possess unique optical properties including strong luminescence, and therefore, their potential for the design of new sensors has been widely recognized [70].

Xiao et al. [73] reported recently a paper-based microarray for multiplexed detection of Hg(II), Pb(II), and Cu(II) based on colorimetric measurements using a smartphone-based sensing system. With this approach, reported results can be generated and shared via wireless network. For this purpose, three kinds of CDs with different fluorescent characteristics were immobilized onto the cellulose substrate to develop a microarray chip. The presence of metal ions gave rise to a decrease in fluorescence of CDs. LODs of 5.8 nM (Hg), 0.12 µM (Pb), and 0.076 µM (Cu) were obtained.

Ngoc Anh et al. [74] developed a paper strip coated with N,S-codoped GQDs for sensing Hg(II) in wastewater. The quantum yield of N,S-GQDs reached 41.9%. Hg(II) quenched the fluorescence of N,S-GQDs, and an LOD of 0.14 nM (in the assay carried out in solution) was achieved. A rapid screening of Hg in wastewater can be performed after filtration and removal of impurities by solid phase extraction followed by UV irradiation of paper strips. A digital camera was used for measuring the color. Hg(II) can easily interact with S atoms, as a result of soft acid and soft base coordination, which results in fluorescence quenching.

Li et al. [75] reported a paper sensor based on fluorescent CDs for the detection of Ce(III), with a LOD of 0.7 µM. Recognition event caused a ‘turned off’ response. The cellulose acetate paper was immersed in a CQDs solution and dried in order to accomplish the sensor. The emission of the test paper was observed by the naked eye under UV irradiation at 365 nm.

Applications of paper-based sensors for elemental speciation is very scarce. Sari et al. [81] described a novel photoluminescent nanocomposite, namely, molecularly imprinted nanoparticles coupled with graphene quantum dots (GMIN) were added onto nitrocellulose membranes (6 mm diameter). The sensor allowed the determination of tributyltin (TBT) with high selectivity and sensitivity in seawater, reaching a LOD of 0.23 ppt. The method allows to circumvent drawbacks inherent to conventional central instrumentation typically employed for TBT detection such as gas chromatography-mass spectrometry (GC-MS) or gas spectrometry-inductively coupled plasma-mass spectrometry (GC-ICP-MS), which are expensive, time-consuming, requiring sophisticated equipment and high technical skills.

A novel dual-emission ratiometric fluorescence sensor was developed by Wang et al. [83] for the detection of Cu(II). For this, a hybrid system integrated by carboxyl-modified red fluorescent CdTe QDs and amino-functionalized blue fluorescent carbon nanodots (CDs) was designed. Cu causes the quenching of the red fluorescence of CdTe QDs whereas the blue fluorescence of CDs is used as internal standard. In this way, a change in fluorescence color from pink to blue occurs, which can be observed by the naked eye under a UV lamp. A LOD of 0.36 nM Cu is achieved, which is much lower than the maximum contamination level given by the United States Environmental Protection Agency (US EPA) in drinking water (20 µM). The sensor was applied to lake water and tap water. The ratiometric probe can be adapted to a paper-based sensor. The probe was printed using the probe solution as ink, thus facilitating advantages of portability and easy operation and opening the door to Cu detection in biological, chemical, and environmental fields without complex instrumentation.

Nitrogen-doped carbon dots were prepared using a one pot method from urea and EDTA for the parallel sensing of Hg(II) and Cu(II) using assays carried out in both solution and cellulose substrates following an on-off-on strategy [85]. LODs of 6.2 and 2.3 nM for Hg and Cu, respectively, for assays performed in solution were reported. LODs of 0.1 and 50 µM for the assays performed on filter paper and a microfluidic device were observed. Fluorescence was quenched by both Cu and Hg, and the quenched fluorescence is recovered (on) in the presence of ascorbic acid (Hg) and citrate (Cu), which allows to discriminate Hg(II) and Cu(II) ions.

Wang et al. [90] described a paper-based sensor for visual detection of Hg(II) using dual-colored carbon dot ratiometric fluorescent test. Hg(II) caused the quenching of the blue fluorescence. The red fluorescence served the purpose of internal standard. Quenching occurs as a result of aggregation induced by Hg(II) whereas the red fluorescence remains unaffected. Thus, a ratiometric sensing approach provided a LOD of 0.14 nM Hg, which is much lower than the maximum contamination level recommended by US EPA for Hg (10 nM) in drinking water.

A ratiometric fluorescent nanoprobe based on label-free carbon dots was applied to the selective determination of Pb(II) and pyrophosphate (PPi) [91]. Emission bands of CDs at 477 and 651 nm were used for sequential detection of Pb(II) and PPi, with LODs of 0.055 and 0.089 µM, respectively. The fluorescence at 651 nm was quenched by Pb(II), whereas the fluorescence at 477 nm remained constant (change from pink to cyan). When PPi was added, a change from cyan to pink occurred.

Dual-emission carbon dots (blue and red CDs) were applied by Wang et al. [92] for speciation analysis of Pb(II). The fluorescent probe was inkjet-printed on filter paper and the color was measured using a smartphone upon irradiation of paper substrates with a UV lamp. The blue fluorescence (maximum fluorescence emission peak at 452 nm) was quenched by Pb(II) whereas the red fluorescence (maximum fluorescence emission peak at 620 nm) remained unchanged. A LOD of 2.89 and 45.9 nM was obtained when using a luminescence spectrometer and a smartphone, respectively. The sensor allows detection of Pb(II) in drinking water at a concentration below the maximum contamination level established by US EPA.

#### 3.2.3. Metal Nanoclusters

Metal nanoclusters (NCs) are made of Au, Ag, Cu, etc., and possess a size smaller than that of metal nanoparticles (NPs). Unlike metal NPs, metal NCs do not display the surface plasmon resonance effect, but they are strongly luminescent [71].

Yuan et al. [77] described an on-site visual method for Hg detection based on CdSe/ZnS QDs functionalized with HDTC. The quenching effect of Hg on the QDs fluorescence due to the formation of a chelate complex on the surface of QDs provided a LOD of 1 ppb. When the assay was performed on a cellulose substrate upon immobilization of HDTC-QDs, a visual color change from orange to red under a UV lamp was observed depending on the Hg concentration, a LOD of 200 ppb being achieved.

**Figure 5:**
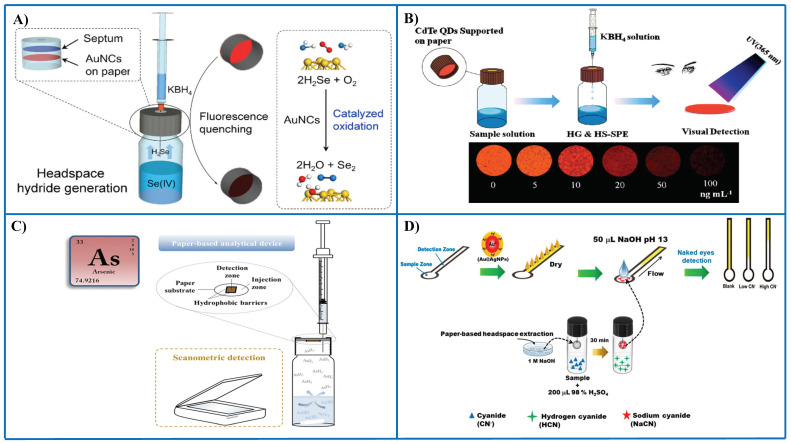
Representative examples of the combination of PADs and thin-film microextraction for the detection of different metal species and anions. (**A**) PAD with AuNCs as fluorescent probes and integrated with thin-film microextraction for sensing Se(IV) following hydride generation [79]; (**B**) PAD with CdTd QDs as nanoreceptors for the determination of Se(IV) following hydride generation [72]; (**C**) PAD integrated with thin-film microextraction for the determination of total As and speciation of As(III) and As(V) [93]; (**D**) distance-based microPAD for the determination of cyanide using Au/Ag NPs as probes; a prior preconcentration in a drop was carried out to improve sensitivity [94]. (**D**). Figure 5A–C is reproduced with permission of the American Chemical Society [72,79] and Elsevier [93].

Xiong et al. [79] described a paper-based assay using fluorescent AuNCs as receptors and hydride generation to convert Se(IV) into SeH_2_ (Figure 5A). AuNCs serve two purposes, i.e., they catalyze the oxidation of SeH_2_ to yield Se(0) that is deposited onto the surface of AuNCs, thus causing fluorescence quenching (recognition event). The separation of Se(IV) as SeH_2_ and its interaction with AuNCs supported paper is carried out inside a vial. An outstanding advantage of this assay is its high tolerance to matrix interferences, overall saline solutions, since a separation is carried out prior to sensing, e.g., 5% m/V salt concentration did not cause any interference effect. An LOD of 4 ppb Se(IV) was achieved with a repeatability around 2.8%. Applications to biological samples and food samples, as well as several waters, proved a suitable performance.

A FRET (Foster resonance energy transfer)-based ratiometric rhodamine immobilized onto CDs was applied for selective Al(III) detection using a paper-based sensor strip [80]. The sensor showed two well-resolved emission peaks, one due to the blue fluorescence of CDs and the other due to the yellow fluorescence of rhodamine moiety. A digital camera was employed to study the changes in emission colors (blue to greenish-yellow) on the paper-based strip. LODs of the assay in solution and on the paper sensor were similar, 3.89 × 10^−5^ M, although the linear range was broader with the paper sensor.

Immobilization of BSA-AuNCs on wax-printed paper was applied to Cu(II) detection [84]. BSA-Au NCs were corroded upon oxidation by H_2_O_2_ in the presence of NH_3_/NH_4_Cl buffer and SCN^-^, which results in fluorescence loss of the probe. When Cu(II) is added, the red fluorescence is kept since the complex Cu(NH_3_)_6_^2+^ can decompose H_2_O_2_. The lost in fluorescence can be related to the Cu(II) concentration in the sample. The potential of this method for field analysis was accomplished by immobilization onto paper. Moreover, typical interferences such as Hg(II) when using AuNCs probes does not occur in this case. Urine, tap water, and sorghum extract were successfully analyzed.

A paper-based sensor along with a smartphone including a home-made image processing algorithm was proposed by Incel et al. [95] for the determination of cyanide. For this, the fluorescent orgametallic dye, europium tetrakis dibenzoylmethide triethyammonium (EuD4TEA) was deposited on the paper surface. Addition of AuNPs caused the quenching of EuD4TEA fluorescence. Upon addition of cyanide solution, a fluorescence recovery and enhancement was observed. Color was processed and the red component was seen to have a linear correlation with cyanide concentration. A LOD as low as 10^−12^ M was achieved. Clear water samples are needed due to interference of colored and crude samples.

Itthiporn et al. [56] described a PAD with enhanced sensitivity for iodate detection by integrating a membraneless gas-separation and a PAD with BSA-AuNCs as sensing probe (Figure 4C). For this, prior reduction of iodate to iodine was performed. The etching of the Au core gives rise to the fluorescence quenching of the probe. Both fluorimetry using a sample holder for the PAD and color measurements under UV illumination following image capture with a smartphone camera are performed. LODs of 0.005 mM (fluorimetry) and 0.01 mM (image capture) are obtained. RSD values were less than 3%. Determination of iodate in fish sauces and iodized salts was successfully carried out.

## 4. Strategies for Enhancing Sensitivity of Paper-Based Analytical Devices

Many PADs and microPADs developed lack the sufficient detection capability for direct detection of metal ions in environmental samples. This is especially a major problem for metals such as Hg, Cd, and to a lesser extent Pb, with maximum contaminant levels in the range of 1–15 ppb (Table 3). It is necessary to highlight that in many applications reported in Table 1 and Table 2, very good LODs are obtained when the assay is carried out in liquid phase with a lab instrument (photometer or fluorimeter), but much worse LODs are observed when the assay is accomplished with image capture. It is generally observed that improved LODs are achieved when applying PADs designs along with nanostructured receptors. In recent years, several strategies have been tried so as to cope with the low concentrations of most metals ions in waters and other environmental samples. In the following section, main strategies attempted for improving sensitivity and LOD along with PADs and microPADs are discussed.

### 4.1. Optimization of PAD and MicroPAD Design

Nguyen et al. [96] have addressed the main shortcomings inherent to the use of microPADs. Liquid suction upon capillary pressure is a fundamental mechanism which explains the flow in two phases (porous network). The main problem is the sample retention and evaporation occurring within the cellulose network, which ultimately limits the transport of analyte to the detection zone and hampers the LOD. Increased performance of microPADs was achieved following the study of variables such travel distances, wax barriers, shape of detection, and sampling zones, etc. For instance, sensitivity was increased by 28% for the detection of Ni(II) when those parameters were optimized.

In relation with PADs, Tan et al. [39] reported a paper-based sensor for determination of Cr(VI) in drinking water. Two shortcomings of PADs are addressed in this paper, i.e., the ‘coffee ring’ effect, which impairs the uniform distribution of the analyte on the cellulose substrate and the high absorbent capacity of common filter paper (Whatman) which facilitates penetration of the sample through the surface, thereby decreasing color intensity. For this, a sensor based on a chemical-responsive adhesive tape was used for improving uniformity of sample distribution on paper. Moreover, the filter paper was subjected to a preliminary superhydrophobic treatment so as to preconcentrate Cr(VI) to a small spot following evaporation of the sample solution. The concentrated spot on the paper surface shows higher reflective intensity as compared to a conventional paper-based sensor. 1,5-diphenylcarbazide was used as a receptor of Cr(VI) upon complexation through the -SH groups. With this approach it is possible to detect Cr(VI) concentration levels as low as 0.05 ppm, thus improving by a factor of 10 the sensitivity of conventional PADs.

However, further improvements of microPADs and PADs design seem to have reached a limit, so new strategies for spreading PADs to the analysis of trace elements in environmental samples are needed. In the following sections, main achievements addressed to improving sensitivity of PADs are discussed.

### 4.2. Preconcentration Strategies in Paper-Based Analytical Devices

#### 4.2.1. Repetitive Deposition of Sample onto the Cellulose Substrate

Most attempts performed with PADs have involved repetitive deposition of sample aliquots for enrichment. Several cycles of deposition/drying are carried out to achieve the suitable sensitivity. The procedure is easy to accomplish but it is labor-intensive and it requires long operation times.

Thus, several metal ions such as Ag(I), Cd(II) Cu(II), Hg(II), etc., have been determined in environmental samples after enrichment, involving prior reaction with pyridilazo receptors [97]. This strategy enables along with the use of chemometric tools the detection of eight elements at microM level. Other PAD using CURNPs as receptors [48] has been used for detection of Hg(II) after 50 accumulation cycles onto the PAD aimed at reaching Hg levels regulated by EPA. Likewise, detection of Hg(II) has been reported using a smartphone and AgNPs as receptor following repetitive accumulation in order to achieve a LOD of 3 ppb Hg [98].

Sharifi et al. [38] have developed 3D origami micro-PADs where a polyvinyl-chloride (PVC) membrane was used to immobilize the chromogenic reagents on the paper surface. This prevents the heterogeneous deposition of the reagents and leaching out the dye due to movement of colored products from the center of the spot to edge of the detection zone, and results in improved sensitivity and LODs. In addition, with the implementation of a waste layer, increased sample volumes can be used. With chrome azurol S and pyrocatechol violet, LODs of 1.7 and 1.9 ppm Cu were achieved along with an extended dynamic range of calibration.

#### 4.2.2. Preconcentration by Solid-Phase Extraction

Cellulosic materials have attracted much interest in recent years, as sorbents for preconcentration and isolation of pollutants in environmental samples. While the presence of hydroxyl groups onto this biopolymer makes it very hydrophilic, these groups also allow many modifications of the cellulose surface. As a result, cellulose-based materials show a great potential as sorbent for several modern preconcentration techniques such as solid-phase extraction (SPE), solid-phase microextraction (SPME), thin-film microextraction (TFME), magnetic-SPE, dispersive-SPME, etc. [99].

Quinn et al. [37] reported a distance-based microPAD as a fast screening tool for Cu monitoring in water systems with a previous preconcentration by solid-phase extraction. A LOD of 20 ppb Cu was obtained and methodology was validated against ICP-MS.

Preconcentration of Pb(II) in water samples has been performed using a cellulose filter containing a sorbent, i.e., Zr silicate [41]. A continuous flow system allows to load the water sample onto the filter in order to achieve a suitable enrichment. Finally the filter is placed in a PAD where Pb(II) comes in contact with the receptor, i.e., a sodium rhodizonate solution, thus allowing a LOD of 10 ppb Pb. In addition, this strategy allows water preservation methods such as pH control, refrigeration, chemical addition, etc., as well as sample storing prior to analysis. The total analysis time including preconcentration and detection was less than 15 min for a 25 mL water sample.

De la Calle et al. [100] described a filtration device on cellulose paper modified with different metal nanoparticles (i.e., AgNPs, PdNPs, AuNPs) for the selective preconcentration of Hg(II). However, in this work, detection on the cellulose substrate was not attempted and Hg was directly measured in vapor phase after complete combustion of the substrate. A preconcentration factor about 3500 and a LOD of 0.2 ppt Hg in waters were achieved.

Zhang et al. [101] reported the determination of Cd(II) in rice after SPE and final detection using a PAD with a fluorogenic reagent, i.e., 4,4-difluoro-4-bora-3a,4a-diaza-sindacene (BODIPY). Fluorescence images of PADs illuminated with UV source light were taken with a digital camera and further digitized with Adobe Photoshop. A LOD of 0.5 µM was obtained, analytical results being comparable to those obtained by inductively coupled plasma-mass spectrometry (ICP-MS).

#### 4.2.3. Preconcentration by Thin-Film Microextraction

CdTe QDs were immobilized on a paper for detection of Se(IV) following hydride generation in a headspace (HS) microextraction system [72] (Figure 5B). Volatile SeH_2_ generated in the vial was extracted in the paper containing CdTe QDs acting as both receptors and preconcentration platforms. Visual detection of Se was accomplished after irradiation at 365 nm in an UV test box. Photoluminescent detection was also performed taking advantage of fluorescent properties of CdTe QDs using a solid sample holder in a fluorescence spectrometer. Since apart from preconcentration a liquid–gas separation occurs, application to complex matrices such as urine can be successfully performed. A LOD of 0.1 ppb Se(IV) with a RSD of 2.4% were reported.

Huang et al. [87] reported the combination of headspace (HS)-TFME and a PAD impregnated with AuNCs for the detection of Zn(II) in biological samples after generation of ZnH_2_ volatile. A LOD of 3 ppb Zn was achieved with a RSD better than 2%. As pointed out above, a paper for the detection of Se(IV) has also been published following hydride generation and TFME onto a PAD impregnated with AuNCs [79].

Bagheri and Saraji [102] have combined headspace single-drop microextraction (HS-SDME) following hydride generation and a paper-based sensor for detection of Se(IV). AuNPs were confined in the drop as receptors for Se(IV) sensing. Thus, analyte separation and preconcentration occurs in the drop containing AuNPs, and additionally, AuNPs serve the purpose of colorimetric probe when spotted onto cellulose paper. The aggregation of AuNPs in the presence of Se(IV) accounts for the redshift of the surface plasmon resonance, color changing from red to blue. This approach allows Se determination in high complexity matrices such as seawater with a quantification limit of 12 ppb.

Pena-Pereira et al. [93] described a paper-based sensor integrated in a microextraction device for As speciation in waters using silver nitrate as receptor (Figure 5C). Both As(III) and As(V) can be determined after conversion of these species in volatile arsine using sodium borohydride as reducing agent. Speciation can be accomplished on the basis of arsine generation at different pH conditions. The reaction between the receptor and the analyte gives rise to the formation of a colored product onto the PAD formed by nano- and microparticles of Ag. The sensor allows also as a screening method for total inorganic As monitoring in waters. An LOD as low as 1.1 ppb As is obtained which is below the maximum contamination level set by US EPA in drinking water. Hydride generation in strong acid media allows arsine generation from As(III) and As(V), but if a weak acid (e.g., citric acid) is employed, only As(III) is reactive toward sodium borohydride. More importantly, this approach brings about several benefits in the development of novel PADs, i.e., no direct contact between the sample and the nanoreceptor occurs, so stability of the sensing surface is not a problem with complex samples such as seawater, wastewaters or complex biological samples; there is a preconcentration on the PAD, thus improving detection capability; it allows speciation analysis by implementing selective reactions in the sample vial.

A similar approach was employed by the authors [103] for the determination of iodide in water samples. Fluorescent CuNCs were employed in drop format under headspace single-drop microextraction configuration and using a PAD. The headspace sampling of iodine generated in situ allows an increase in selectivity and sensitivity for iodide sensing. An enrichment factor of ca. 1100 times was achieved, yielding a LOD of 1 ppb iodide with the drop sensor. Although less sensitive, the PAD can provide on-site sensing using a tablet camera.

Cyanide was determined in several types of waters (drinking water, seawater, tap water, and wastewater) by means of distance-based PAD using Au/Ag NPs as probes [94] (Figure 5D). The yellow band length caused by the probe decreased with increasing cyanide concentration. In order to increase the sensitivity of the assay, a headspace-single drop microextraction approach was implemented. The 30-fold enrichment thus obtained enabled a LOD of 10 ppb cyanide by the naked eye. In other approach, cyanide was determined in water and wastewater by combining a PAD impregnated with pyridine-barbituric acid and headspace extraction [104].

Sulphite was determined in food samples by combining a headspace-thin film microextraction with a PAD impregnated with Fe(III) + 1,10-phenanthroline [57] (Figure 4D). Sulfite was converted into SO_2_ in the vial after acidification, which caused the reduction of the Fe(III) to Fe(II), and in turn, the development of a red color in the PAD due to the complex formed between Fe(II) and the chromogenic reagent. A smartphone camera was employed for capturing the color image, providing a LOD of 0.04 ppb.

A headspace sampling device combined with a PAD as sensing platform for SO_2_ determination in wine has been described by Li et al. [105]. For this, 4-mercaptopyridine (Mpy)-modified Au nanorods (AuNRs)-reduced graphene oxide hybrids, anhydrous methanol, and starch-iodine complex were employed as probes. Both colorimetric and surface-enhanced Raman scattering (SERS) were applied as dual detection modes. Following the Karl Fischer reaction, the deep-blue color due to the formation of a complex between starch and iodine results in a decrease in color and an increase of the SERS signal. The combined HS-PAD strategy allows an efficient separation and preconcentration of SO_2_. Concentrations of 5 and 1 µM can be detected by the naked eye and SERS.

A dual PAD was described by the authors [106] for the simultaneous detection of nitrite and sulphide in waters. For this, a PAD containing two separate sections drawn with a permanent marker was built. The probes, i.e., Griess reagent and Cu(II) solution, were deposited in each separate section of the PAD, which was placed in the screw cap of a 40 mL vial for headspace sampling. Selective volatilization reactions were implemented in the vial yielding nitrogen oxides and hydrogen sulphide from nitrite and sulphide, respectively. LODs of 5 and 28 ppb were achieved for nitrite and suphide, respectively. The PAD integrated within headspace microextraction shows an improved sensitivity (8 and 124 enhancement for nitrite and sulphide, respectively) as compared to most PADs and microPADs described in the literature.

## 5. Conclusions and Outlook

Unlike analytical techniques available in central labs, paper-based analytical devices (PADs and microPADs) allow low sample and reagent consumption, portability, easy operation, and real time measurements. Thus, in the environmental field, these analytical systems can successfully meet several requirements, such as easy access to remote sites, preliminary evaluation of water quality, spatial and temporal variability of pollutants aimed at determining their sources, distribution, and environmental impact. On the other hand, there exists a demand for disposable sensors for field applications in resource-limited regions. The spread use of disposable sensors could facilitate to take actions in a fast way in order to assess contamination events in different socioeconomic contexts.

The current state of paper-based analytical devices in environmental analysis allows to conclude that although several works have been reported on detection of some transition (e.g., Cu, Ni, Co, etc.) and heavy metals (Hg, Cd), there is a lack for other elements of high toxicity (e.g., As, Sb, Tl, Bi, etc.). The availability of PADs and microPADS for chemical speciation (e.g., valence states of As, Se, Sb, etc.) is also practically null. In addition, scarce attention has been paid to toxic organometals of environmental relevance such as methyl-Hg, tetraethyl-Pb, organic-Mn, etc. Likewise, there is still a small number of assays based on PADs for the detection of anions (with exception of nitrite). As was mentioned previously, environmental applications have been limited to samples with simple matrix, but there is a lack of assays for samples with high contents in salts, organic matter, etc.

Finally, other shortcomings identified in this review include (i) difficulties of many PADs and microPADs to cope with the low concentration levels of relevant toxic elements (e.g., Hg) in order to comply with international regulations for drinking water; (ii) almost absence of applications of PADs to the detection of trace metals in seawater; (iii) few assays available for multielemental analysis. Sensing approaches based on PADs with nanostructured receptors provide, in general, better analytical performance (sensitivity and LOD) as compared to traditional chromogenic/fluorogenic reagents. Implementation of nanomaterials with enhanced optical properties and integration of the cellulose-based sensing platforms with novel microseparation strategies could lead to opening up new avenues so as to expand the scope of paper-based sensors for trace element analysis and speciation in the environmental field. In this sense, characteristics such as sensitivity, selectivity, precision, multiplexing ability, robustness, and stability should be focused on and, therefore, new developments are expected in the future.

## Figures and Tables

**Table 1:** Applications of colorimetric detection of metal ions using PADs and µPADs along with chromogenic/fluorogenic and nanostructured receptors.

Target Analyte	Sample/Matrix	Nanostructured Receptor	Sensor Design	Signal Acquisition	Limit of Detection	Ref.
***Chromogenic/fluorogenic reagents***						
Cs(I)	Waters	Chrysoidine	PAD	Mobile camera	100 ppb	[25]
Cu(II)	Drinking water	Porphirin derivative	µPAD	Distance-based measurement (naked eye)	1 ppm	[26]
Cu(II), Cd(II)	Waters	Diethyldithiocarbamate,	3D µPAD	Mobile phone	0.29 (Cu), 0.33 (Ni)	[27]
Ni(II), Cr(VI)		dimethylglyoxime, cadion, diphenylcarbazide			0.19 (Cd), 0.35 (Cr) (in ppm)	
Cr(VI)	Airborne particulate	Diphenylcarbazide	µPAD	Desktop scanner	0.12 µg Cr	[28]
Ni(II), Cr(VI),	Lake water	Dimethylglyoxime,	Rotational µPAD	Hand-held device	4.8 (Ni), 1.6 (Cu)	[29]
Cu(II)	Seawater	diphenylcarbazide, bathocuproine		(Reflectance)	0.18 (Cr) (in ppm)	
Mn(II), Co(II)	---	PAR	µPAD	Naked eye (no quantitative)	Screening of masking agents	[30]
Cu(II)	Tap and river water	*S*-Nitroso-N-acetylpenicillamine (SNAP) (catalytic effect)	PAD	Mobile camera	1.2 µM	[31]
Hg(II), Cu(II)	Tap and lake	Zincon, dimethylglyoxime,	µPAD	Digital camera,	1 ppb (Hg), 20 ppb (Cu)	[32]
Cr(VI), Ni(II)	water	diethyldithiocarbamate, diphenylcarbazide		scanner	0.15 ppm Cr(VI), 0.23 ppm Ni(II).	
Cu(II), Ni(II), Fe(III)	Airborne particulatematter	Dimethylglyoxime, dithiooxamide, bathophenanthroline,	µPAD (distance-based)	Scanner	0.1 (Ni, Cu) 0.05 (Cr) (in µg)	[33]
Fe(III), Cu(II), Ni(II)	Particulate matter	1,10-phenanthroline, dimethylglyoxime, bathocuproine	µPAD	Scanner	1.5 (Fe), 1 (Cu) 1 (Ni) (in µg)	[34]
Ni, Fe, Cu, Cr	Particulate matter	1,10-phenanthroline, dimethylglyoxime, diphenylcarbazide, bathocuproine	µPAD	Scanner	0.75 (Fe, Ni, Cu), 0.12 (Cr) (in µg)	[35]
Cu(II), Fe(III), Zn(II)	Waters	Zincon, bathophenanthroline, dithizone	µPAD	Distance-based	100 ppb (ca. 1-2.5 ppb with previous membrane preconcentration	[36]
Cu(II)	Drinking water	Dithiooxamide	µPAD SPE preconcentration	(distance-based)	(20 ppb)	[37]
Cu(II)	Rain and tap water	Chrome azurol S and pyrocatechol violet	3D µPAD	Scanner	1.7 and 1.9 ppm, resp.	[38]
Cr(VI)	Tap water	1,5-difenylcarbazide	PAD (SH-CAT)	Scanner	0.05 ppm Cr(VI)	[39]
Fe(II), nitrite	Tap water	Griess (nitrite),	PAD	Reflectance	0.53 ppm (nitrite)	[40]
BSA, glucose		1,10-phenanthroline (Fe)		spectroscopy		
Pb(II)	Drinking water, tap water, wastewater	Sodium rhodizonate	µPAD	Smartphone, scanner	10 ppb Pb	[41]
Fe(III), Ni(II)	River water	1,10-phenanthroline,	µPAD	Tablet camera	0.1 mM (Fe), 0.5 mM (Ni)	[42]
BSA		dimethylglyoxime, tetrabromophenyl blue			and (BSA) (µM)	
Hg(II)	--	Ir(III) complex	PAD	Naked eye	1.88 × 10^−3^ M	[43]
B(III)	Wastewater, Seawater	Curcumin, *Curcumin Longa* L. extracts	PAD	Scanner, tablet camera	0.2-0.8 ppm (depending on PAD)	[44]
***Nanostructured receptors***						
Cr(VI)	River water	BSA capped-AuNPs	Silanization-TiO_2_ Modified paper	Scanner	280 nM	[45]
Cu(II)	Pond, tap water Hg(II)	AgNPs Pond, river water	PAD and µPAD AuNPs PAD	Digital camera, smartphone	0.5 ppb Cu 50 nM	[46][47]
Hg(II)	Pond and tap water	PtNPs and TMB	PAD	Digital camera and smartphone	0.01 µM	[48]
Hg(II)	Waters	Curcumin NPs	PAD	Digital camera	0.17 ppm (direct) 0.003 ppm (Prec.)	[49]
Hg(II), NH_3_	---	AgNPs (phytosynthesis)	PAD	Photometry and naked eye	5 ppb Hg, 5 ppm NH_3_ (Photometry), 5 ppm (naked eye)	[50]
Ni(II)	River water Tap water	Zincon-ZnSiO_3_ nanospheres	PAD	Scanner	36 nM Ni	[51]
Fe(III)	Water, blood	AgNPs-CTAB	PAD	Smartphone	20 ppb Fe	[52]
Ag(I), Cu(II) Hg(II)	Water	ZnSe nanocrystals	PAD	Mobile camera	1 ppm (Cu) 5 ppm (Hg, Ag)	[53]
Pb(II), Cu(II)	Waters	AuNPs-TA-DNS	µPAD	Naked eye	>10 ppb	[54]
Cu(II)	River water	ZnO/ZnS core shell NPs	PAD	Digital camera	15 µM	[55]

**Table 2:** Applications of the detection of metal ions by PADs and microPADs using luminescent nanomaterials as receptors.

Target Analyte	Sample/Matrix	Nanostructured Receptor	Sensor Design	Signal Acquisition	Analytical Characteristics	Ref.
Se(IV)	Hair, sediment, urine	CdTe QDs	PAD	Fluorescence under UV irradiation	0.1 ppb Se	[72]
Hg(II), Pb(II), Cu(II)	River water	CDs	PAD (microarray)	Smartphone	5.8 nM (Hg) 0.12 µM (Pb) 0.076 µM (Cu)	[73]
Hg(II)	Wastewater	GQDs (N, S-codoped)	PAD	Digital camera (UV irradiation)	0.14 nM	[74]
Ce(III)	River water	Carbon dots	PAD	Naked eye (UV irradiation)	0.7 µM (fluorimetry)	[75]
Fe(III)	--	N-doped GQDs	PAD	Naked eye (UV irradiation)	2.37 µM (fluorimetry)	[76]
Hg(II)	Water, urine	CdSe/ZnS QDs (functionalized with HDTC)	PAD	Naked eye (UV irradiation)	0.2 ppm Hg	[77]
Ag(I), AgNPs	River water	CdTe QDs	PAD	Naked eye (UV irradiation, distance measurement)	0.01 ppm (Ag)	[78]
Se(IV)	Tap water, seawater fish, rice, eggs	AuNCs	PAD	Naked eye (UV irradiation) and fluorimetry	4 ppb (Se)	[79]
Al(III)	----	C-dots-R6G	PAD	Digital camera (UV irradiation) and fluorimetry	3.89 × 10^−5^ M (PAD)	[80]
Tri-butyl-Sn	Seawater	GQDs conjugated with MIN	PAD	Fluorimetry	0.23 ppt	[81]
Hg(II), Cu(II)	Tap and river water	CS-CDs	Sponge cellulose	Mobile camera (UV irradiation)	26 nM (Al); 0.11 and 3 µM (Cu), by fluorimetry and PAD, resp.	[82]
Cu(II)	Tap water, Lake water	Hybrid CdTe-CDs	PAD	Digital camera (UV irradiation)	0.36 nM (fluorimetry)	[83]
Cu(II)	Urine, tap water, Sorghum extract	BSA-AuNCs	PAD	Naked eye (UV irradiation)	5 nM (fluorimetry)	[84]
Cu(II), Hg(II)	Tap water	N-doped CDs	µPAD	Digital camera (UV irradiation)	0.1 µM (µPAD); 6.2 nM (Hg), 2.3 nM (Cu) by fluorimetry	[85]
Cu(II), Hg(II)	Tap and lake water	CQDs	PAD	Naked eye	5 (Cu), 3 (Hg) (µM)	[86]
Zn(II)	Water, blood, cells	AuNCS	PAD	Naked eye and fluorimetry	3 ppb (fluorimetry) 20 ppb (naked eye under UV irradiation)	[87]
Co(II)	---	ZnS QDs	PAD	Mobile camera	10 ppm	[88]
Cu(II)	--	ZnO NPs	PAD	Mobile camera	10 µM	[89]
Hg(II)	Tap water Lake water	CDs	PAD	Naked eye (UV irradiation)	0.14 µM	[90]
Pb(II)	---	CDs	PAD	Fluorimetry and naked eye	0.055 µM (fluorimetry)	[91]
Pb(II)	Waters	CDs	PAD	Fluorimetry and smartphone camera	2.89 nM (fluorimetry) 45.9 nM (PAD)	[92]

**Table 3:** Concentration values of metal ions and anionic species regulated by EPA, WHO, and European Directive in drinking waters.

Chemical Species	EPA ^a^	WHO ^b^	European Directive ^c^
Al	--	--	200 ppb
As	10 ppb	10 ppb	10 ppb
Sb	6 ppb	20 ppb	5 ppb
Ba	2 ppm	1.3 ppm	
Be	4 ppb		
Cd	5 ppb	3 ppb	5 ppb
Cr(T)	100 ppb	50 ppb	50 ppb
Cu	TT * action level = 1.3 ppm	2 ppm	2 ppm
Pb	TT * action level = 15 ppb	10	10 ppb
Hg	2 ppb	6 ppb	1 ppb
Se	50 ppb	40 ppb	10 ppb
Tl	2 ppb		
Ni	--	70 ppb	20 ppb
B	--	2.4 ppm	1 ppm
U	--	30 ppb	
Fe	--	--	200 ppb
Mn	--	--	50 ppb
Cyanide	200 ppb		50 ppb
Fluoride	4 ppm	1.5 ppm	1.5 ppm
Nitrate	10 ppm	50 ppm	50 ppm
Nitrite	1 ppm	3 ppm	0.5 ppm
Bromate	10 ppb	10 ppb	10 ppb
Chlorite	1 ppm	0.7 ppm	
Chlorate	--	0.7 ppm	
Chloride			250 ppm
Sulphate	--	--	250 ppm

^a^ Maximum contaminant level ^b^ Guideline value ^c^ Parametric value * TT: treatment technique.

## Data Availability

No new data were created or analyzed in this study. Data sharing is not applicable to this article.

## Abbreviations

AgNPs	silver nanoparticles
AuNCs	gold nanoclusters
AuNPs	gold nanoparticles
AuNRs	gold nanorods
BODIPY	4,4-difluoro-4-bora-3a,4a-diaza-sindacene
BSA	bovine serum albumin
CDs	carbon dots
CQDs	carbon quantum dots
CTAB	cetyltrimethylammonium bromide
CuNCs	copper nanoclusters
CURNs	curcumin nanoparticles
DNA	deoxyribonucleic acid
EDTA	ethylenediaminetetraacetic acid
EuD4TEA	europium tetrakis dibenzoylmethide triethyammonium
FRET	Foster resonance energy transfer
GC-MS	gas chromatography-mass spectrometry
GMIN	molecularly imprinted nanoparticles coupled with graphene quantum dots
GQD	graphene quantum dots
HDTC	2-hydroxyethyldithiocarbamate
HS	headspace
ICP-MS	inductively coupled plasma-mass spectrometry
LOD	limit of detection
LSPR	localized surface plasmon resonance
NCs	nanoclusters
NPs	nanoparticles
NRs	nanorods
PADs	paper-based assay devices
microPADs	microfluidic paper-based devices
PAR	4-(2-pyridylazo) resorcinol
PDMS	polydimethylxilosane
PdNPs	palladium nanoparticles
PtNPs	platinum nanoparticles
QDs	quantum dots
RSD	relative standard deviation
RSNO	S-nitrosothiol
SDME	single-drop microextraction
SERS	surface-enhanced Raman scattering
SH-CAT	superhydrofobic chemical-responsive adhesive tape
SNAP	S-Nitroso-N-acetylpenicillamine
SPE	solid-phase extraction
SPME	solid-phase microextraction
SPR	surface plasmon resonance
STCP	silanization-titanium dioxide modified filter paper
ssDNA	single strand DNA
TAS	total analysis systems
TA-DNS	thioctic acid-dansylhydrazine
TBT	tributhyltin
TFME	thin-film microextraction
TICs	information and communication technologies
TMB	3,3,5,5-tetramethylbenzidine
US EPA	United States Environmental Protection Agency
WHO	World Health Organization
